# Ethical Views on Sharing Digital Data for Public Health Surveillance: Analysis of Survey Data Among Patients

**DOI:** 10.3389/fdata.2022.871236

**Published:** 2022-04-25

**Authors:** Renee Garett, Sean D. Young

**Affiliations:** ^1^ElevateU, Irvine, CA, United States; ^2^Department of Emergency Medicine, University of California, Irvine, Irvine, CA, United States; ^3^Department of Informatics, University of California Institute for Prediction Technology, University of California, Irvine, Irvine, CA, United States

**Keywords:** data sharing, ethics, social media, digital data, privacy

## Abstract

Digital data, including social media, wearable device data, electronic health records, and internet search data, are increasingly being integrated into public health research and policy. Because of the current issues around public distrust of science and other ethical issues in public health research, it is essential that researchers conduct ongoing studies assessing people's perceptions around and willingness to share digital data. This study aims to examine participants' social media use and comfort sharing their data with health researchers. One hundred and sixty-one participants with medical conditions were recruited through social media paid advertisements and referral from a website, and invited to complete surveys on social media use and ethical perspectives on data sharing. Eligibility criteria were adults 18 years old or older, living in the US, self-reported having been diagnosed by a physician with a medical condition, belonging to at least one social media platform, using social media at least twice a week, and owning a smartphone. Study participants were mostly female, White, and with a mean age of 36.31 years. More than one third of participants reported being very comfortable sharing electronic health data and social media data for personalized healthcare and to help others. Findings suggest that participants are very uncomfortable sharing their location and text message data with researchers, with primary concerns centered around loss of privacy, disclosing private information, and that friends, family, and others may find out that they shared text messages with researchers. We discuss the implications of this research before and after the COVID-19 pandemic, along with its potential implications for future collection of digital data for public health.

## Introduction

Investigators have utilized digital tools such as social media, electronic health records (EHR), and global positioning system (GPS) data for a variety of purposes in research. Social media technologies have been used to recruit participants, collect and mine data, and conduct interventions (Young et al., 2015; Dol et al., 2019). Research topics on this subject have varied including infectious diseases, substance use, cancer, mental health, and chronic conditions (Dol et al., 2019; Garett and Young, 2021c). For the most part, participant reception and acceptance of social media use by academic researchers have been positive, though concerns about privacy and anonymity, risk of harm, and effects on vulnerable populations were present (Golder et al., 2017). Studies examining the acceptance of EHR use indicate that people perceive it will improve their healthcare (Dimitropoulos et al., 2011; Entzeridou et al., 2018). Similar to social media data, participants' surveyed have expressed concerns such as privacy and that the information would be used to discriminate against them (Entzeridou et al., 2018).

Despite concerns about security and privacy loss, study participants have generally agreed that benefits such as improved medical care and quality of care for them and others outweighed the risks (Dimitropoulos et al., 2011). With respect to location data investigators have used GPS in research in various ways including examining children (Mizen et al., 2020) and adult mobility (Megges et al., 2018). Feasibility and acceptance studies, even among marginalized populations, indicate that participants may be highly comfortable with researchers tracking their location (Duncan et al., 2020). However, similar to social media data and EHR, participants have expressed concerns with privacy and confidentiality and risk of harm (Mirzazadeh et al., 2014). Because of people's growing mistrust of science, the spread of misinformation, and the importance of incorporating proper ethical procedures into research (Golder et al., 2017; Garett and Young, 2021a,b, 2022), it is essential that researchers continue to assess public perceptions on and willingness to share digital data for use in public health. It is especially important to study this topic during and after the COVID-19 pandemic, as digital surveillance and outreach methods are increasingly being used.

Among individuals with diagnosed medical conditions, this study aims to examine participants' social media use and comfort/concerns with sharing their data with health researchers.

## Methods

### Recruitment

Participants were recruited through Facebook advertisements or through referral from Moshemu.com, a website for posting research studies. Facebook advertisements ran from February through March of 2020. Interested individuals who clicked on the Facebook advertisements were routed to an online interest form for initial screening. Screening questions included contact information, age, medical diagnoses, type and frequency of social media use, and ownership of a smartphone. Research staff called participants to verify information and obtain informed consent. Moshemu website staff provided study research staff with a list of participants meeting eligibility criteria. Moshemu participants were emailed the study information sheet by study staff. Those who replied expressing interest in the study were called to verify information and obtain verbal consent. Eligibility criteria were adults 18 years old or older, living in the US, self-reported having been diagnosed by a physician with a medical condition, belonging to at least one social media platform, using social media at least twice a week, and owning a smartphone. All eligible participants were emailed a link to the online survey.

The online survey took ~15 min to complete and included items related to type of social media and other digital technology platforms used; frequency of use; comfort/perceptions of ethical views on sharing data related to these platforms, including EHR, social media, mobile app, and mobility/GPS data; as well as views about sharing these data with researchers and/or industry. Participants received $15 in online gift cards as compensation.

### Data Analysis

Of the 163 respondents, 161 participants had completed the survey in full and were included in the analysis. Descriptive analysis characterized demographics and social media and smartphone use, as well as comfort level and concerns about sharing private information. Correlation analysis assessed associations between variables. All analyses were performed using SPSS v25.

The study was approved by the University Institutional Review Board (IRB#18-002043).

## Results

Participant demographics are shown in Table 1. Study participants were mostly female (*n* = 97), White (*n* = 101), and with a mean age of 36.31 years (range18–70 years). Education and social media use were fairly distributed across the participant sample. The top three medical diagnoses were mental health (*n* = 83), chronic pain (*n* = 36), and autoimmune disease (*n* = 19). Table 1 also shows participant behaviors and preferences on social media. The three most-frequently used social media platforms were Facebook (*n* = 159), Instagram (*n* = 131), and Twitter (*n* = 97). The top three methods of messaging were Facebook Messenger (*n* = 142), text messages/SMS (*n* = 137), and iMessage (*n* = 73).

**Table 1:** Participant demographics, social media use, and smartphone use.

**Characteristics**	***N* (%)**
**Gender**	
Female	97 (60.2)
Male	56 (34.8)
Non-binary	2 (1.2)
Transgender	6 (3.7)
**Race/ethnicity**	
American Indian or Alaskan native	3 (1.9)
Asian	34 (21.1)
Black or African American	14 (8.7)
Hispanic, Latino, or Spanish origin	4 (2.5)
White	101 (62.7)
Mix/other	4 (2.5)
**Age**	
18–25	42 (26.1)
26–35	49 (30.4)
36–45	31 (19.3)
46–55	21 (13.0)
56–65	14 (8.7)
66+	4 (2.5)
**Education**	
< High school	6 (3.7)
High school/GED	42 (26.1)
Associate's degree/vocational training/some college	36 (22.4)
College graduate	57 (35.4)
Graduate school	20 (12.4)
**Medical condition** ^**a**^	
HIV/AIDS	9 (5.6)
Mental health	83 (51.6)
Substance use disorder	11 (6.8)
Autoimmune disease	19 (11.8)
Cardiovascular disease	10 (6.2)
Chronic pain	36 (22.4)
Cancer	6 (3.7)
Diabetes	31 (19.3)
Other	58 (36.0)
**Hours per day on social media**	
0–1	4 (2.5)
1–2	26 (16.1)
2–3	47 (29.2)
3–5	35 (21.7)
5+	49 (30.4)
**Social media platforms** ^**a**^	
Facebook	159 (98.8)
Twitter	97 (60.2)
LinkedIn	92 (57.1)
Snapchat	75 (46.6)
Instagram	131 (81.4)
Reddit	61 (37.9)
Venmo	69 (42.9)
TikTok	31 (19.3)
Other	3 (1.9)
**Texting platform** ^**a**^	
Facebook Messenger	142 (88.2)
WhatsApp	46 (28.6)
Google Hangouts	24 (14.9)
Text messages (SMS)	137 (85.1)
iMessage	73 (45.3)
Other	7 (4.3)

a *Participants were asked to select all items that applied to them*.

As depicted in Table 2, more than one third of participants reported being very comfortable sharing electronic health data for personalized healthcare (*n* = 64) and to help others (*n* = 54). Fewer than 5% of participants were very uncomfortable sharing data for personalized healthcare (*n* = 7) or to help others (*n* = 6). Similarly, more than one third of respondents reported being very comfortable sharing social media data for personalized healthcare (*n* = 55) and to help others (*n* = 51), while fewer than 10% of participants were very uncomfortable with sharing data for personalized healthcare (*n* = 12) or to help others (*n* = 16). Less than one third of participants felt comfortable sharing their GPS location and text message/smartphone data with researchers for both personalized health care (*n* = 48) and to help others (*n* = 42), with almost a quarter of participants reportedly very uncomfortable sharing their GPS location and text message data for personalized healthcare (*n* = 39) or to help others (*n* = 36).

**Table 2:** Participant concerns and comfort level sharing data with researchers.

**Type of data shared with researchers**	**Purpose of research**	
	**Personalized healthcare for participant**	**Greater public health**
	***N* (%)**	***N* (%)**
**Electronic health record** ^**a**^		
Very uncomfortable	7 (4.3)	6 (3.7)
Uncomfortable	11 (6.8)	20 (12.4)
Neutral	44 (27.3)	42 (26.1)
Comfortable	35 (21.7)	39 (24.2)
Very comfortable	64 (39.8)	54 (33.5)
**Social media data** ^**a**^		
Very uncomfortable	12 (7.5)	16 (9.9)
Uncomfortable	19 (11.8)	16 (9.9)
Neutral	32 (19.9)	35 (21.7)
Comfortable	43 (26.7)	43 (26.7)
Very comfortable	55 (34.2)	51 (31.7)
**GPS location and text message data** ^**a**^		
Very uncomfortable	39 (24.2)	36 (22.4)
Uncomfortable	26 (16.1)	29 (18.0)
Neutral	21 (13.0)	28 (17.4)
Comfortable	27 (16.8)	26 (16.1)
Very comfortable	48 (29.8)	42 (26.1)
**Concerns**	**Data source**	
	**Social media** ***N*** **(%)**	**Smartphone (including GPS data)** ***N*** **(%)**
**Concerned that data provided to research team would be used in ways that might be harmful**		
Not at all concerned	25 (15.5)	27 (16.8)
Somewhat concerned	50 (31.1)	38 (23.6)
Neutral	21 (13.0)	19 (11.8)
Concerned	34 (21.1)	40 (24.8)
Very concerned	31 (19.3)	37 (23.0)
**Concerns in providing health researchers access to data** ^**b**^		
Disclosing private information	116 (72.0)	120 (74.5)
Might affect health insurance coverage	38 (23.6)	30 (18.6)
Friends, family, or others might find out	67 (41.6)	60 (37.3)
Might affect my medical care	35 (21.7)	33 (20.5)
Loss of privacy	114 (70.8)	121 (75.2)
No concern	7 (4.3)	4 (2.5)
Other	7 (4.3)	5 (3.1)

a *Comfort level for type of data shared for personalized healthcare and to help others correlated with each other p < 0.01*.

b *Participants were asked to select all items that applied to them*.

Participants were also asked about their concern with sharing social media and smartphone data with researchers. 19.3% (*n* = 31) of respondents were very concerned that providing social media data to researchers could harm them, 31.1% (*n* = 50) were somewhat concerned, 13.0% (*n* = 21) were neutral, 21.1% were concerned (*n* = 34), and 15.5% (*n* = 25) were not at all concerned. A similar distribution was seen for participants' concern sharing smartphone data, including GPS location data, with researchers, with 23.0% (*n* = 37) very concerned, 24.8% (*n* = 40) were somewhat concerned, 11.8% (*n* = 19) were neutral, 23.6% were concerned (*n* = 38), and 16.8% (*n* = 27) were not at all concerned. The top three reasons why participants were concerned about sharing their social media data to researchers were disclosing private information (*n* = 116), loss of privacy (*n* = 114), and friends, family, or others might find out (*n* = 67). As for sharing smartphone data, including GPS locations, the top three concerns were loss of privacy (*n* = 121), disclosing private information (*n* = 120), and friends, family, and others might find out (*n* = 60). Less than 5% of participants reported having no concern sharing social media data (*n* = 7) or smartphone data (*n* = 4).

Participants' comfort level in sharing electronic health record data for personalized healthcare was highly correlated with sharing social media data (*r* = 0.78, *p* < 0.01) and sharing GPS location and text message data (*r* = 0.90, *p* < 0.01). Participants' concern for sharing social media data was significantly associated with concerns about the following issues: disclosing private information, concerns that sharing data might affect health insurance coverage, concerns that sharing data might affect medical care, loss of privacy, and concern in sharing smartphone/GPS data (all, *p* < 0.01).

## Discussion

This study examined social media use among individuals with a medical diagnosis as well as their comfort level and the concerns they have with sharing private data with researchers. Study results show that the majority of participants currently have a Facebook account, followed by Instagram and Twitter. Our result echoed findings by other investigators whose patient samples had a higher number of Facebook account over other social media platforms (Timms et al., 2014; Ashford et al., 2018). Moreover, our study sample reported that they were generally comfortable sharing social media data to researchers. With respect to electronic health record, our results support findings by Weitzman et al. (2010) and Stevenson et al. (2013). Which show comfort in sharing EHR records for research purposes. Results also indicate a correlation between research for personalized healthcare and to help others which suggest that regardless of who benefits from the information, individuals with a medical diagnosis are comfortable sharing data for science. However, EHR and social media data highly correlated with each other, but not with GPS location which could indicate that there are some data that participants are less comfortable sharing even if it benefits them.

To our knowledge, this is the first study to report on the types of concern individuals with medical diagnosis have regarding sharing their GPS location and text message data with researchers. Although previous studies have shown acceptance of researchers collecting location data among individuals with a medical diagnosis (Liu et al., 2017), none have explored specific concerns regarding sharing GPS data for research purposes. Our findings indicate that more participants are very uncomfortable sharing their location and text message data with researchers than their EHR or social media data, with primary concerns centered around loss of privacy, disclosing private information, and that friends, family, and others may find out that they shared text messages with researchers. This discomfort may be due to the type of behavior they exhibit on social media vs. their smartphone. Participants reported liking a post, reading other's posts, or viewing others' videos and images more frequently than sharing their own content on social media (data not shown). Smartphone data, such as location and text messages, are more private and personal compared to social media posts on public forums and they may not be comfortable sharing that to others.

Interestingly, people do not appear to have consistent ethical views/perceptions about sharing digital data. In fact, individuals in this study were surveyed ~1 month prior to the introduction of the COVID-19 pandemic in the United States. Approximately one month after the pandemic began, we followed up with participants from this study who reported being willing/very willing and unwilling/very unwilling to share their digital data for use in public health. Not surprisingly, those who were already willing to share data continued to be willing to share data. However, a surprising effect was that after the start of the pandemic, more than 75% of these participants who initially reported being unwilling to share their digital data for public health now reported being willing to share their data (Romero and Young, 2022). This underscores the need for ongoing research on people's views on sharing digital data, as well as additional research into the factors that affect people's willingness and change over time.

This study has limitations. Recruitment was limited to advertising on Facebook and a research website designed to recruit research participants, potentially leading to a biased sample. This could also impact generalization of findings as it might not generalize beyond individuals recruited in this manner. However, it is likely that this type of recruitment method would have broad reach (1) given that ~70% of people use Facebook^1^, and (2) because this type of online recruitment has become very standard in health research and is continues to grow as use of the internet continues to grow (Singh et al., 2020; Garett and Young, 2021b). Another limitation is the sample composition, which is mostly white women. There may be a sex and ethnic bias with regard to privacy issues regarding sharing data with researchers that were not explored given our sample. Furthermore, our sample also consisted of mostly participants under 56 years of age. It would be of interest to determine how comfortable elderly adults are with sharing their smartphone data given that 79% own and use them (Pew Research Center, 2019).

Although the use of digital tools is well-established, investigators may want to emphasize the purpose of the research, who will have access to the data, and the safety protocols for privacy to assuage participant concerns. Outside of ethical considerations inherent in human research, transparency with participants about the nature of data collection and steps used to maximize privacy cannot be overstated. Likewise, acknowledging and understanding the concerns of participants could help shape research methodology and protocols. Individuals with medical diagnosis are comfortable sharing their data to assist researchers in developing personalized healthcare as well as to benefit others. Future research efforts could focus on marginalized populations, such as the transgender communities, to explore their specific concerns and comfort level sharing medical records, social media, and location data, and how they feel it would affect their privacy and medical care. Use of new technologies and privacy perspectives continue to evolve to prevent the growing COVID-19 pandemic, making this research especially timely and important to learn whether and how participants would be willing to share data to assist in controlling the pandemic.

## Data Availability Statement

The original contributions presented in the study are included in the article/supplementary materials, further inquiries can be directed to the corresponding authors.

## Ethics Statement

The studies involving human participants were reviewed and approved by UCI IRB. The patients/participants consented online through the use of an online written information sheet.

## Author Contributions

RG and SY developed the concept and edited manuscript drafts. Both authors contributed to the article and approved the submitted version.

## Funding

This work was supported in part by the National Institute of Allergy and Infectious Diseases under Grant number 7R01AI132030, the National Center on Complementary and Integrative Health (NCCIH), and the National Institute on Drug Abuse (NIDA).

## Conflict of Interest

RG is the PI of a NIH SBIR-funded study for the website, moshemu, that was used for participant recruitment in this study. The remaining author declares that the research was conducted in the absence of any commercial or financial relationships that could be construed as a potential conflict of interest.

## Publisher's Note

All claims expressed in this article are solely those of the authors and do not necessarily represent those of their affiliated organizations, or those of the publisher, the editors and the reviewers. Any product that may be evaluated in this article, or claim that may be made by its manufacturer, is not guaranteed or endorsed by the publisher.

## Footnotes

^1^NW 1615 L. St, Suite 800Washington, Inquiries D 20036USA202-419-4300 | M-857-8562 | F-419-4372 | M. Demographics of Social Media Users and Adoption in the United States [online]. Pew Research Center: Internet, Science and Tech. Accessed online at: https://www.pewresearch.org/internet/fact-sheet/social-media/ (accessed: May 21, 2020).
